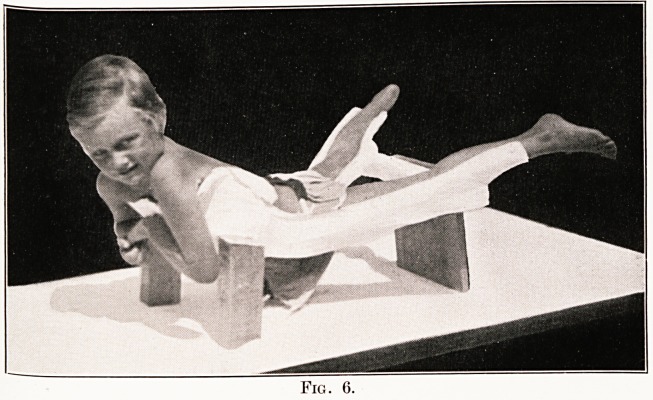# The Use of the Ventral Position in the Treatment of Tuberculous Disease of the Spine in Children
*Reprinted by permission from the Annual Report for 1932 of the Medical Officer of Health for Bristol.


**Published:** 1933

**Authors:** K. H. Pridie

**Affiliations:** Medical Superintendent, Frenchay Park Sanatorium and Orthopædic Hospital, and Assistant Orthopædic Surgeon to the Bristol City Council


### THE USE OF THE VENTRAL POSITION IN THE TREATMENT OF TUBERCULOUS DISEASE OF THE SPINE IN CHILDREN.*

BY

K. H. Pridie, M.B., B.S., F.R.C.S.,

*Medical Superintendent, Frenchay Park Sanatorium and Orthopædic Hospital, and Assistant Orthopædic Surgeon to the Bristol City Council.*

---

It is very difficult to discuss the treatment of tuberculous disease of the spine, as the treatment is so lengthy and the results so distant and so easily modified by the future home and social conditions of the patient.

It is easy to produce destructive criticism of the accepted methods of treatment, but very hard to suggest any treatment which is an improvement on the standard methods.

The disadvantages of the treatment of cases by some modification of the double abduction spinal frame can be considered under the following headings :—

1. General condition and comfort of the child.
2. The muscular system.
3. The skeletal changes.

* Reprinted by permission from the Annual Report for 1932 of the Medical Officer of Health for Bristol.

1. The child treated on a double abduction frame can do very little for itself, its education is very limited, the direct line of vision and, consequently, its outlook on life is limited to a white ceiling or a blue sky. Its external surroundings have to be viewed through a mirror or after contortions of the neck. In the supine position mental concentration is difficult and reading becomes very tiresome, writing for any length of time an impossibility.

2. One or two years' treatment on a spinal frame causes a marked generalized wasting of the muscles of the body, especially those of the erector spinæ group. The skin becomes slightly œdematous over the outer and posterior parts of the thigh and trunk. During the cold weather the efforts of the child to draw the legs together to keep warm tends to produce knock-knee. In spite of the constant efforts of the masseuse the leg muscles waste. Great care has to be taken in the nursing of these cases to prevent hyper-extension of the knees, foot-drop, stiff knees and hips.

In many cases the disuse atrophy is so extreme that the convalescent period has to be prolonged, the time being spent in muscular development and re-education, and finally some form of posterior spinal brace has to be worn to take the place of the atrophied posterior spinal muscles.

3. Prolonged treatment with absolute immobilization causes marked decalcification of all the bones of the body; added to this there is the local decalcification due to the tuberculous lesion.

PLATE IX

FIG. 1.

FIG. 2.

FIG. 3.

This marked decalcification, attended by an increased excretion of calcium from the body, seems to predispose to renal calculi.

There is often a marked flattening of the chest from before backwards in those children that have been treated in spinal frames for several years. This is accompanied by a lateral spreading of the ribs, which gives the patient a very peculiar appearance. This must be due to the weight of the body on the very decalcified ribs.

In order to overcome these defects of the frame treatment I have been treating cases of tuberculous disease of the spine in children where the disease has been situated in spinal segments between the mid-dorsal and lower lumbar regions in the prone position in plaster hyper-extension beds.

*The method of construction of the plaster beds.*

The child is placed on an orthopædic table as shown in Fig. 1. The front of the abdomen and legs are coated with ung. zinci et ol. ricini. One layer of plaster bandages is applied to the front of the child, the rest of the case is made from pressed slabs of plaster, which are applied till the whole case is about $\frac{3}{8}$ in. thick. This is removed and cut to the correct shape. The feet are then fixed on the cast. The inside is polished till it is smooth. The case is then dried and afterwards varnished with cellulose varnish.

If the case is carefully made no padding is needed, in fact the children are more comfortable without it. The plaster beds are screwed to the galvanized iron top of the ordinary beds.

*Important points in the construction of the plaster beds.*

The legs are abducted, the knees slightly bent to prevent the knee ever becoming fully extended. The child is secured to the bed by a bandage round each thigh and round the lumbar region. From the knee downwards the child can move the legs as much as he pleases. There can be no tendency to develop drop-foot, knock-knee or hyper-extension of the knee.

A certain movement of the spine is possible in the direction of increased hyper-extension, the plaster prevents flexion and lateral movement. The constant muscular effort of lifting the head and shoulders develops the posterior spinal muscles to an extraordinary degree, and these muscles are more efficient than any spinal brace during the convalescent stage.

Figs. 2 and 3 show a plaster bed as it is when finished. Slight modifications are made when the lesion is in the mid-dorsal region; in these cases a head rest is made, as shown in Fig. 4. When the lesion is situated in the lumbar region no head-rest is fitted.

Figs. 5 and 6 show the patients in the plaster beds; a small bed table is provided in front of the child, on which the patient can rest his arms, and on which two pillows are put for the head to rest at night.

*Duration of treatment in plaster beds.*

When the disease appears to be quiescent, as shown by temperature chart, radiological examination,

PLATE X

Fig. 4.

Fig. 5.

Fig. 6.

and good compensating curves have developed, the child is taken out of the plaster bed and treated in the prone position during the day, with a triangular pillow under the upper part of the trunk, to maintain the hyper-extension of the spine. During the night they are allowed to sleep on their backs. During the periods when children are free in bed exercises are performed every day in the form of leg movements.

This trial period lasts at least three months, and should the child progress satisfactorily, it is then allowed crawling exercises, and finally to walk.

During the convalescent period the child is either in the prone position or walking. Great care is taken to prevent the child sitting or standing about. They attend school, and are taught in the prone position on canvas stretchers.

### Conclusion.

Comparing my series of cases, about half of which have been treated in the prone position as outlined above, and the other half in the dorsal position in double abduction frames, I find :

1. The children are happier in the prone position, they eat better, do the school work better, and need less attention.

2. Their general muscular tone is splendid. Their compensating curves develop much more quickly, there is no stiffness of the knees, limited movement, or weak ligaments, even after two years of treatment.

There is not such marked generalized decalcification as one finds after even short periods of frame treatment.

3. The average duration of treatment in uncomplicated cases is reduced by one-third.

4. No spinal brace has to be worn.

## Figures and Tables

**Fig. 1. f1:**
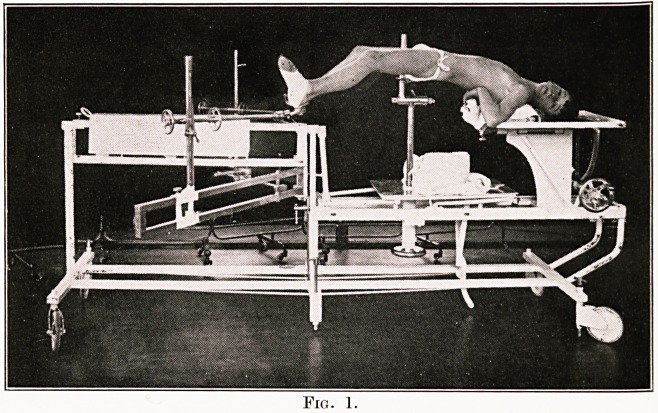


**Fig. 2. f2:**
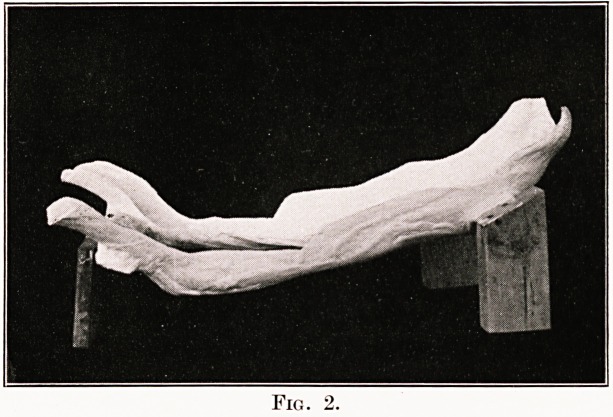


**Fig. 3. f3:**
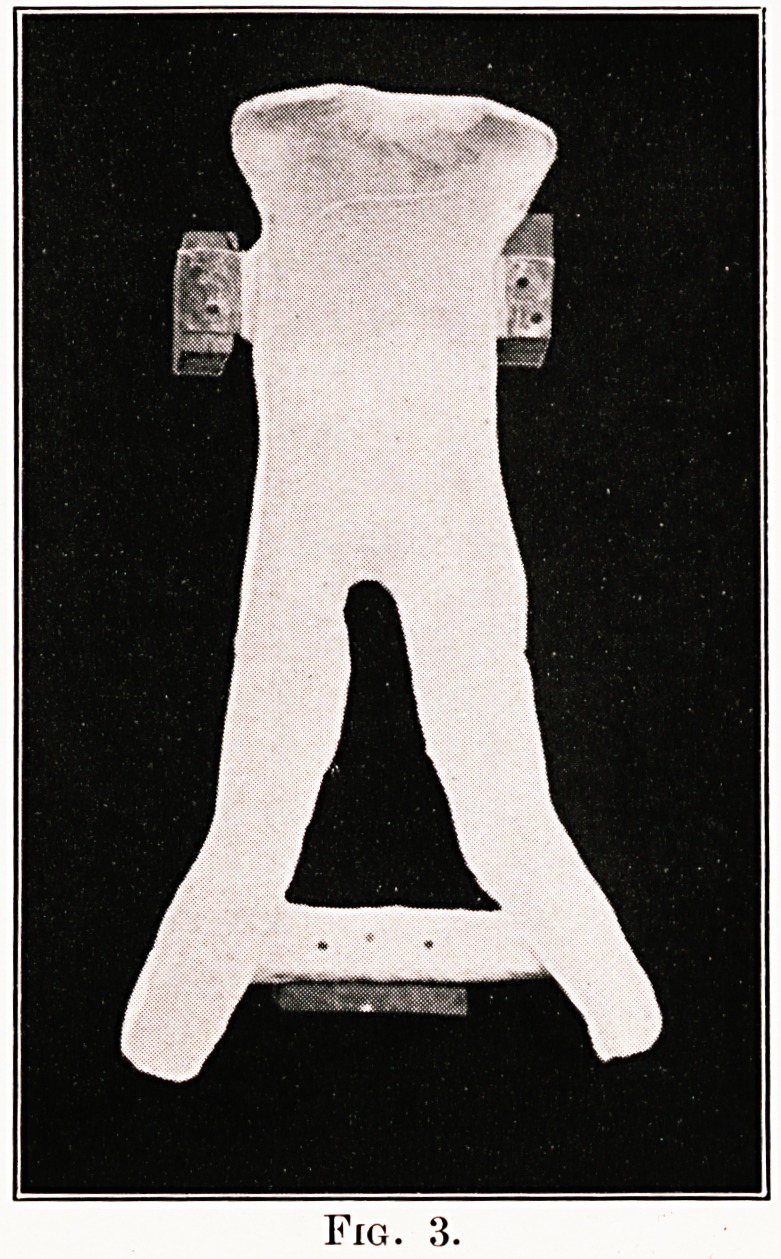


**Fig. 4. f4:**
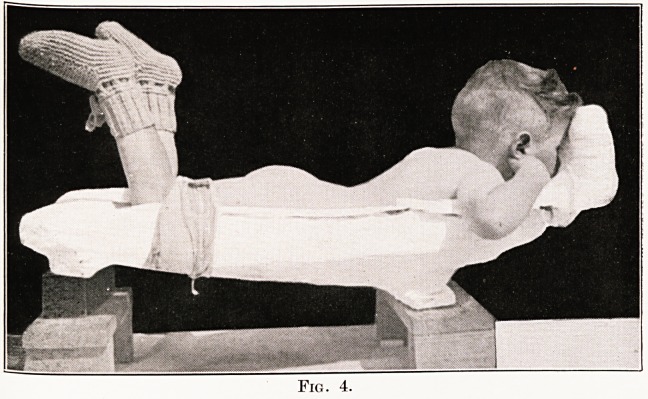


**Fig. 5. f5:**
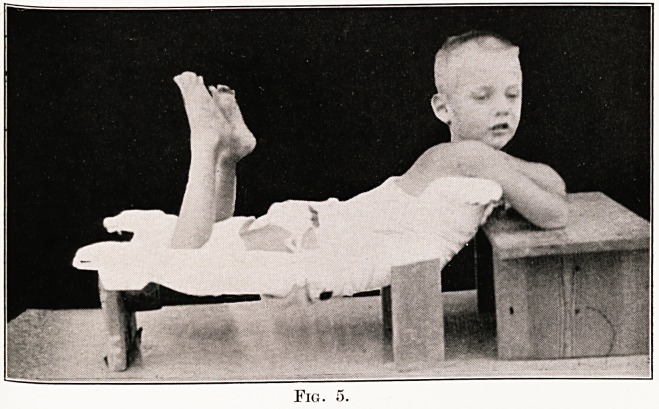


**Fig. 6. f6:**